# Role of frataxin protein deficiency and metabolic dysfunction in Friedreich ataxia, an autosomal recessive mitochondrial disease

**DOI:** 10.1042/NS20180060

**Published:** 2018-11-02

**Authors:** Elisia Clark, Joseph Johnson, Yi Na Dong, Elizabeth Mercado-Ayon, Nathan Warren, Mattieu Zhai, Emily McMillan, Amy Salovin, Hong Lin, David R. Lynch

**Affiliations:** 1 Department of Neurology, University of Pennsylvania School of Medicine, Philadelphia, PA, U.S.A.; 2 Division of Neurology, Children’s Hospital of Philadelphia, Philadelphia, PA, U.S.A.; 3 Penn/CHOP Center of Excellence in Friedreich Ataxia, Philadelphia, PA, U.S.A.

**Keywords:** ataxia, biogenesis, reacrive oxygen specie

## Abstract

Friedreich ataxia (FRDA) is a progressive neurodegenerative disease with developmental features caused by a genetic deficiency of frataxin, a small, nuclear-encoded mitochondrial protein. Frataxin deficiency leads to impairment of iron–sulphur cluster synthesis, and consequently, ATP production abnormalities. Based on the involvement of such processes in FRDA, initial pathophysiological hypotheses focused on reactive oxygen species (ROS) production as a key component of the mechanism. With further study, a variety of other events appear to be involved, including abnormalities of mitochondrially related metabolism and dysfunction in mitochondrial biogenesis. Consequently, present therapies focus not only on free radical damage, but also on control of metabolic abnormalities and correction of mitochondrial biogenesis. Understanding the multitude of abnormalities in FRDA thus offers possibilities for treatment of this disorder.

Friedreich ataxia (FRDA) is an autosomal recessive, neurodegenerative disorder that affects roughly 1 in every 50–100000 people in the United States. FRDA was first described in 1863 as a disease that is primarily early onset, associated with progressive limb and gait ataxia, absent tendon reflexes from the legs, axonal sensory neuropathy, dysarthria, muscle weakness, spasticity in the lower limbs, and loss of position and vibration sense [1–4] (Table 1). Neurodegeneration occurs early in the large proprioceptive sensory neurones of the dorsal root ganglia (DRG) and their axons in the posterior columns, with later atrophy of the corticospinal and spinocerebellar tracts of the spinal cord and the dentate nucleus in the cerebellum [5–9]. There is also loss of pancreatic islet cells and hypertrophic cardiomyopathy, which is the most common cause of death amongst FRDA patients. Patients can also develop scoliosis (curvature of the spine), pes cavus (fixed plantar foot flexion; severely high-arched feet), hearing loss (from auditory neuropathy), and vision loss (from optic neuropathy) [9–12]. In addition, fatigue is a dominating symptom amongst people with FRDA.

**Table 1:** Clinical features of FRDA

System	Pathology	Clinical result
Neurological	Degeneration of large sensory neurones – proprioception	Loss of balance and coordination
		Loss of deep tendon reflexes
	Degeneration of spinocerebellar tracts (dorsal)	Loss of balance and coordination
	Degeneration of dentate nucleus of the cerebellum	Loss of balance and coordination
		Dysarthria (slurred speech)
		Eye movement abnormalities (modest)
	Degeneration of corticospinal tracts	Spasticity, pyramidal weakness
Visual	Degeneration of retinal ganglion cells	Optic neuropathy
Auditory	Degeneration of auditory nerve	Auditory neuropathy
Cardiac	Hypertrophic cardiomyopathy, with early hypertrophy, later fibrosis	ECG abnormalities
		Arrhythmias
		Progressive heart failure
Endocrine	Loss of pancreatic islet cells	Diabetes mellitus
	Increased insulin resistance	Diabetes mellitus
Orthopedic	Scoliosis	
	Pes cavus (fixed plantar foot flexion; high arched feet)	

Abbreviation: ECG, electrocardiogram.

FRDA results from decreased levels of functional frataxin protein, coded by the *FXN* gene on chromosome 9 [13,14]. Such decreases in frataxin levels are caused by guanine-adenine-adenine (GAA) trinucleotide repeats within intron 1 of the *FXN* gene in the vast majority of abnormal alleles. In patients carrying two expanded alleles (96%) in FRDA patients, the length of the allele with the shorter GAA expansion inversely correlates with frataxin levels, age of onset, and rate of disease progression; longer alleles result in earlier onset and faster progression [15–18]. A subset of FRDA patients have GAA expansion in one chromosome and a point mutation in the *FXN* exon in the other chromosome [19–22]. Most point mutations lead to absence of frataxin production by alterations in the start codon, RNA splice sites, or in residues needed for protein folding. Other mutations do not lower protein levels but instead appear to disrupt the function of frataxin.

Expanded GAA repeats may form unusual triplex structures, disrupting RNA polymerase and preventing transcription elongation [23]. In addition, epigenetic mechanisms decrease frataxin expression as regions flanking GAA repeat expansion exhibit marks of condensed heterochromatin. There is also increased methylation of specific CpG sites, reduction in histone H3 and H4 acetylation levels, and increased histone H3 lysine 9 (H3K9) trimethylation in FRDA lymphoblasts, peripheral blood, brain, and heart [24–28]. Overall, this leads to a decrease in *frataxin* mRNA synthesis and a decrease (but not absence) in frataxin protein in people with FRDA [29–32]. As the phenotype of FRDA in subjects with point mutations altering frataxin production or stability is almost identical with those with GAA repeats, the clinical syndrome largely if not entirely reflects the loss of frataxin protein rather than the effects on *frataxin* mRNA levels.

## Frataxin protein structure, function, and role in metabolism

FRDA patients’ peripheral tissues typically have less than 10% of the frataxin levels exhibited by unaffected people, and the level of frataxin inversely correlates with disease severity [29–32]. The *FXN* gene contains seven exons (exons 1–4, 5a, 5b and 6), with exons 4 and 5a being the most conserved across species [33]. *Frataxin* mRNA is translated by cytoplasmic ribosomes and translocated to the mitochondria based on an N-terminal mitochondrial localization sequence. Upon entry into the mitochondria, frataxin undergoes a two-step proteolytic cleavage by mitochondria processing peptidase (MPP) to generate the mature protein [34–36]. The mature protein forms a twisted, six-stranded β-antiparallel sheet, flanked by N- and C-terminal α helices (α1 and α2) [37]. The negatively charged residues on the helical plane may bind iron, while the uncharged residues on the surface β sheet can lead to protein–protein interactions [38].

Frataxin functions in iron metabolism, iron storage, and iron–sulphur cluster biosynthesis, with resultant effects on many downstream events [39–43] (Table 2). A conserved primary Fe^2+^-binding site, with a dissociation constant within the micromolar range (3–55 μM), is contained in residues of the acidic ridge in the first α helix. In addition to iron binding, frataxin interacts with mitochondrial aconitase, ferrochelatase, and proteins of the mitochondrial Fe–S cluster synthesis pathway [44]. Iron and Fe–S clusters are essential for metabolic processes including electron transport, DNA synthesis, both redox and non-redox reactions, as well as other cellular functions [45,46]. Iron–sulphur containing proteins play a crucial role in cellular respiration and ATP production; therefore, decreased activity should significantly impair mitochondrial function. Frataxin’s role in iron–sulphur cluster biogenesis makes it essential for enzymatic activity of Fe–S containing aconitase and respiratory chain complexes. Consequently, decreased frataxin levels result in decreased aconitase activity in cell culture models, *in vivo*, and in heart tissues and biopsies of FRDA patients [47,48]. These effects on key enzymes of energy production lead to a failure of ATP production in FRDA, as observed in humans in muscle spectroscopy [49–51]. This may represent one of the more important pathophysiological events in FRDA, as it is clearly observable in human muscle in FRDA, and is readily linked to one of the most important symptoms of FRDA, fatigue.

**Table 2:** Selected cellular functions of frataxin

Protein	Function
Isu1/Nfs1	Scaffold proteins for Fe–S biogenesis. Frataxin controls iron entry and sulphur production through activation of cysteine desulphurization
Aconitase	FXN facilitates and stabilizes transfer of Fe group to Aconitase to convert it into its active form
Ferrochelatase	FXN meditates iron delivery to Ferrochelatase in heme synthesis
Succinate dehydrogenase	FXN regulates entry of electrons into Complex II of electron transport chain
ATP synthase	FXN regulates entry of electrons into Complex II of electron transport chain. Reduced FXN expression is correlated to a reduction in ATP
Pyruvate dehydrogenase	Pyruvate dehydrogenase subunit E3 may exhibit proteolytic activity capable of cleaving FXN under certain conditions
p38	FXN deficiency may alter p38 mitogen-activated protein kinase signaling
Nrf2	FXN deficiency impairs Nrf2 translocation to the nucleus
Nitric oxide	NO increases as a result of FXN deficiency. This increase is related to the increase in ROS due to iron accumulation. NO increases as a protective effect from Fe-mediated oxidative stress
PGC1α	PGCα is the master regulator of mitochondrial biogenesis. FXN deficiency results in dysregulation of PGC1α. This is tissue dependent but is down-regulated in most cell types
PDK1	Frataxin deficiency triggers the activation of PDK1 through increasing phosphorylation levels of S241 and may deactivate pyruvate dehydrogenase and decrease cell metabolism
Iron uptake, import, and export protein	Frataxin deficiency causes increased expression of transferrin receptor 1 and mitochondrial iron importer mitoferrin-2, and decreased expression of ferroportin1, contributing to increased iron accumulation in mitochondria

Abbreviations: Nrf2, nuclear factor E2-related factor 2, PGC1α, peroxisome proliferator-activated receptor γ coactivator 1-α.

Additionally, frataxin deficiency may secondarily affect enzymes of intermediary metabolism. In addition to direct effects on iron–sulphur cluster-containing enzymes, specific cellular and mitochondrial enzymes are regulated through frataxin level or the resultant effects on ATP levels. For example, while FRDA patients have normal pyruvate dehydrogenase (PDH) activity in most tissues [52], under certain conditions, including mitochondrial acidification, the dehydrogenase subunit (E3) of PDH exhibits proteolytic activity that is capable of cleaving frataxin [53]. Although PDH is likely not the only enzyme controlled by frataxin levels, it provides an example of how enzyme-specific regulation at the intersection of multiple mitochondrial metabolic pathways could control cellular phenotype through alteration of metabolism. FRDA patient platelets exhibit significantly decreased acetyl Co-A (Ac-CoA) synthesized through glycolysis than healthy control platelets [54,55] while creating substantially more Ac-CoA and HMG-CoA from palmitate. This emphasizes how the collection of changes in Fe–S containing enzymes alter flux through specific pathways. Recent evidence additionally suggests that frataxin deficiency may alter p38 kinase signaling, providing further evidence of a role for frataxin in signaling and metabolism [56]. Thus, the alterations in Fe–S containing and other enzymes provide a manner for specific frataxin-related changes in metabolism, which may have deleterious effects on cells.

## Frataxin deficiency and mitochondrial dysfunction

**Figure 1:**
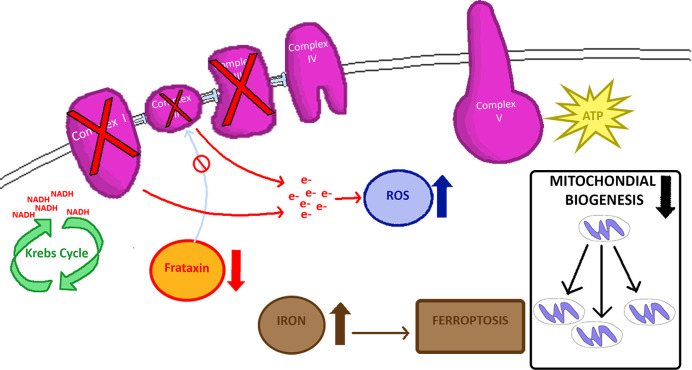
Mitochondrial features of FRDA Frataxin deficiency leads to loss of Fe–S groups in Complexes I, II, III with downstream ROS production and other downstream events.

Frataxin overexpression demonstrates this protein’s crucial role in mitochondrial energy conversion and oxidative phosphorylation (OXPHOS), as well as regulation of the Krebs cycle [57] (Figure 1). Frataxin directly interacts with Complex II subunits, suggesting it directly supports the electron transport chain by providing Fe–S complexes [58–60]. Endomyocardial biopsies of FRDA patients exhibit decreased Complexes I, II, and III activity [61], and FRDA mouse models demonstrate mitochondrial biogenesis impairment and OXPHOS dysfunction in respiratory chain complexes I, II, and IV in cerebellum [62].

Frataxin deficiency is also linked to mitochondrial dysfunction through iron accumulation and production of reactive oxygen species (ROS). Although produced throughout the cell, 90% of ROS result from mitochondrial respiration. During the transfer of electrons from the mitochondrial respiratory chain to molecular oxygen (O_2_) in OXPHOS, a small percentage of electrons will leak, resulting in the production of ROS, such as hydroxyl (HO^−^) and hydrogen peroxide (H_2_O_2_) [63–65]. This leak mainly occurs at Complexes I and II [66]; however, when ROS levels rise too high, oxidative damage, also termed as oxidative stress, can occur in the cell, especially in mitochondria. Oxidative stress damages proteins and DNA, especially mtDNA, as mtDNA lacks the protection from histones and the complex nuclear repair system [66]. Oxidative stress induces apoptosis by opening the mitochondrial permeability transition pore, and has been implicated in a number of neurodegenerative diseases, such as Parkinson’s disease (PD), Alzheimer’s disease (AD), amyotrophic lateral sclerosis, and multiple sclerosis [67,68].

ROS production occurs in multiple models of FRDA [69–73]. In certain *Drosophila* models with induced frataxin deficiency, H_2_O_2_-scavenging enzymes ameliorate features of oxidative stress and restore both ROS-sensitive mitochondrial enzymes and aconitase activities to normal levels. These findings implicate H_2_O_2_ as a pathogenic mediator of ROS production in FRDA and suggest that H_2_O_2_-scavenging molecules could play a therapeutic role in treating the disease [64]. In fibroblasts from patients with FRDA, treatment with iron-containing compounds or hydrogen peroxide leads to oxidative stress, activation of caspase 3, and apoptosis [74–76]. Analogous results have been identified across many cell types, and treatment with many proposed antioxidant-based therapies restores the healthy phenotypes [77,78]. Consequently, oxidant-induced cell death remains an area of interest for possible FRDA therapies.

One proposed component of increased ROS sensitivity in FRDA patient cells is the accumulation of mitochondrial iron [79–84]. Based on Fenton chemistry, mitochondrial iron accumulation has the potential to dramatically increase susceptibility to ROS [84]. However, FRDA involves iron maldistribution more than iron overload; cells behave as if they are depleted of iron cytosolically while simultaneously having a mitochondrial iron overload [85–87]. Systemic iron indices such as ferritin levels are normal to low in most FRDA patient tissues, except for the heart, where ferritin excess is noted at autopsy [88]. This raises the possibility that the direct effect of iron in FRDA may be tissue-specific.

The components of ROS production and iron overload are combined in a paradigm of cell death referred to as ferroptosis. Ferroptosis is a form of iron-dependent, oxidation-mediated, programmed cell death implicated in a variety of pathological processes, including neurotoxicity, neuroinflammation, and neurodegenerative diseases such as PD, AD, and ischemic stroke [89–92]. Ferroptosis may share some of the same downstream signaling pathways as apoptosis, but this form of cell death differs from classical apoptosis, and the mechanisms that underlie ferroptosis match many of the abnormal findings of FRDA [89–92]. Upon induction of ferroptosis, there is a lack of morphological or biochemical features of apoptosis, such as chromatin condensation and nuclear shrinkage [89,93]. Additionally, there is no cleavage-mediated activation of caspase 3, and caspase inhibitors do not protect against ferroptosis [89]. Oxidative stress releases iron from ferritin in a redox active form, induces lipid peroxidation, particularly of polyunsaturated fatty acids, and leads to accumulation of lipid-based ROS [89,93,94]. Accumulation of lipid peroxidation products and ROS derived from iron metabolism triggers ferroptosis as a response to these harmful metabolic events [92]. Ferroptosis may also be triggered following depletion of intracellular reduced-glutathione (GSH) levels, further leading to increased cellular availability of iron as a ferroptosis catalyst [91].

In addition to ROS generation, ferroptosis is associated with the loss of mitochondrial integrity [89–92]. EM shows cells treated with ferroptosis inducers exhibit obvious changes in mitochondrial morphology [89]. Investigators have found that a protein originally characterized during pro-apoptotic signaling, BID, translocates to the mitochondria during ferroptotic signaling. BID can act as a sensor of oxidative stress in an iron-dependent manner and its translocation to mitochondria mediates the loss of mitochondrial integrity and function [90]. Induced ferroptosis in neurones leads to loss of mitochondrial membrane potential, increased mitochondrial fragmentation, reduced ATP levels, and permeabilization of the outer mitochondrial membrane [90]. Distinct morphological alterations are also apparent, including decreased mitochondrial size, condensed mitochondrial membranes, reduction in mitochondrial cristae, and outer mitochondrial membrane rupture [90–92].

Lipid peroxidation, elevated ROS generation, GSH depletion, and increased iron availability are all pathogenic alterations found in many neurologic diseases, and interestingly, they are also common features of ferroptosis [91]. The dysregulated iron metabolism of FRDA suggests that ferroptosis may also play a role in cell death in FRDA.

ROS production is difficult to demonstrate in humans with FRDA. Although some studies find elevated urinary oxidative stress biomarker levels, specifically the isoprostanes dihydroguanosine and malondialdehyde, isoprostanes are not elevated in FRDA and only a single study has found abnormalities in DNA oxidation [95–99]. Moreover, confounding factors, including the overwhelming use of antioxidant supplements by FRDA patients and the relative inactivity of such patients leading to a lack of ongoing OXPHOS and an absence of ROS, result in further challenges to demonstrate ROS accumulation in FRDA patients [98]. It is also possible, however, that significantly increased ROS production is not continually occurring in FRDA. Not all cell death in animal models of FRDA is associated with detectable ROS production or iron accumulation. In mouse models of FRDA, cell death occurs without detectable accumulation of ROS or iron [100]. Such data provide evidence that in these models, other events such as loss of specific enzymatic activities, failure of ATP production, or other processes may be sufficient to induce cell death in FRDA without inducing ferroptotic pathways.

## Failure of nuclear factor E2-related factor 2 and mitochondrial biogenesis pathways

Nuclear factor E2-related factor 2 (Nrf2) is a transcription factor that regulates cellular antioxidant response under oxidative stress conditions. Under normal conditions, the interaction between Nrf2 and Keap1 leads to the degradation of Nrf2 through the ubiquitin-proteasome pathway [101]. Typical oxidative stress conditions inhibit the interaction between Nrf2 and Keap1, leading to increased levels of active Nrf2 [102,103]; however, Nrf2 is degraded in FRDA patients and laboratory models, which is unexpected in an environment of oxidative stress [102,104].

In the presence of ROS, Nrf2 induces the expression of ROS-response antioxidant genes such as heme oxygenase-1 (HO-1), NAD(P)H quinone oxidoreductase 1 (NQO1), Cu/Zn and Mn-superoxide dismutases (SOD 1,2), glutathione synthetic enzymes, and others by binding to the antioxidant response element (ARE) on nuclear DNA, including an ARE site within FXN [104,105]. In a healthy state, oxidative stress causes Nrf2 translocation to the nucleus, resulting in the expression of antioxidant genes to protect cells from damage. In FRDA models, Nrf2 translocation to the nucleus is compromised in response to oxidative insults, thus leading to reduced expression of antioxidant genes such as *NQO1* and *SOD-1,2* [101,106]. This may increase vulnerability to oxidative stress and lead to a cascade of oxidant-induced damage in neurons and other cell types. Interestingly, studies to find compounds that induce Nrf2 lead to identifying compounds that up-regulate frataxin gene expression [101]. Thus, Nrf2 expression correlates with frataxin expression. Nrf2 also regulates synthesis of GSH, a tripeptide antioxidant that moderates ROS production and ferroptosis [107]. In FRDA, the altered homeostasis between reduced and oxidized glutathione, increases cells’ susceptibility to oxidative stress [62,104,107].

In addition to increased ROS production and paradoxical loss of Nrf-2, frataxin deficiency is also associated with other components of mitochondrial dysfunction in both FRDA patients and animal models. Mitochondrial biogenesis deficits appear in multiple models of FRDA, including human lymphocytes and mouse models such as the frataxin knockin/knockout (KIKO) mouse [108–110]. Interestingly, the levels of PGC-1a, the master regulator of mitochondrial biogenesis, are significantly decreased in cerebellar homogenates of KIKO mice, even when mice are behaviorally asymptomatic [62]. This suggests early impairment of mitochondrial biogenesis pathways as a potential mediator of mitochondrial loss and dysfunction in FRDA. Parallel dysfunction in downstream genes of the entire PGC-1a/NRF1/Tfam pathway in KIKO mouse cerebellum confirms mitochondrial biogenesis impairment as an early event in this model.

Other markers of mitochondrial number fusion are also altered in FRDA. The mitochondrial chaperone, glucose-related protein 75 (GRP75), which physically interacts with frataxin, and the mitochondrial fusion protein mitofusin-1 (MFN1), are lower in cerebellar homogenates of FRDA KIKO mice [62]. Human FRDA fibroblast and PBMCs also show decreased GRP75 levels [111,112]. Furthermore, in KIKO mice, this decrease is associated with a long-term deficit in mitochondrial number, suggesting that in some brain regions, FRDA may give rise not only to abnormal mitochondria, but also lead to decrease in numbers of mitochondria [62]. Although the correlation between GRP75 levels and the severity of FRDA remains to be determined, GRP75 reduction should result in further decreases in frataxin levels and iron–sulphur cluster biogenesis and may thus impact mitochondrial function. Alternatively, GRP75 reduction could potentially lead to mtDNA damage, thereby contributing to the progression of FRDA.

## Clinical trials and therapeutic strategies

At present, there is no cure or effective treatment for FRDA [113]. Current strategies aim to increase frataxin expression or target downstream pathways affected secondary to frataxin deficiency [114–120]. High-throughput screening with different cellular models is also being used to search for new drugs. Even when restorative therapies for frataxin achieve success, mitochondria-based therapies are still likely to be useful covering the deficiencies in restoration of frataxin levels.

### Antioxidants and OXPHOS

Frataxin deficiency potentiates cellular damage from oxidative stress, suggesting that antioxidants might present a therapeutic approach for FRDA. For example, idebenone is a short-chain Coenzyme Q_10_ (CoQ_10_) analog that acts as an antioxidant by protecting membrane lipids from peroxidation and stimulating OXPHOS and ATP production by carrying electrons from Complexes I and II to Complex III in the electron transport chain [121]. Initial enthusiasm for idebenone was highly based on its ability to protect respiratory Complex II from iron inactivation and decreased lipoperoxidation; however, neither idebenone nor other similar agents have proven effective in double-blind trials as compared with placebo [122–125]. Other antioxidants like CoQ_10_ with vitamin E, and VP20629 have also shown no benefit in clinical trials [126].

### Iron chelating strategy

As the pathogenesis of FRDA involves an imbalance in the intracellular accumulation of iron, with mitochondrial accumulation and relative cytosolic depletion, targetted iron chelation could be beneficial in restoring a healthy iron balance. Deferiprone, an iron chelator that localizes to the mitochondria, rapidly distributes in the CNS, crossing membranes, and can penetrate mitochondria to remove excess iron [127]. Deferiprone has a lower affinity for iron than other iron chelators (pFe^3+^ log stability constant of 19.9 compared with deferoxamine (26.6) and less tendency to cause overall iron depletion, leading to an improved safety profile over other iron chelators in patients with low iron overload [128]. It restores mitochondrial redox potential, reduces ROS, and increases aconitase activity, without affecting frataxin levels [129–133]. The drug is typically well tolerated and can be administered orally. However, exacerbation of tremor occurred at high doses and the risk of agranulocytosis remains a threat of deferiprone treatment [133].

## Abbreviations

Ac-CoA: acetyl Co-A
AD: Alzheimer’s disease
ARE: antioxidant response element
CoQ_10_: coenzyme Q_10_
FRDA: Friedreich ataxia
GAA: guanine-adenine-adenine
GRP: glucose related protein
GSH: glutathione
KIKO: knockin/knockout
Nrf2: nuclear factor E2-related factor 2
NQO1: NAD(P)H quinone oxidoreductase 1
OXPHOS: oxidative phosphorylation
PD: Parkinson’s disease
PDH: pyruvate dehydrogenase
ROS: reactive oxygen species
SOD: superoxide dismutase
